# Electrochemical Properties and Structure of Multi-Ferrocenyl Phosphorus Thioesters

**DOI:** 10.3390/molecules25040939

**Published:** 2020-02-19

**Authors:** Ruslan Shekurov, Mikhail Khrizanforov, Tatiana Gerasimova, Zilya Yamaleeva, Kamil Ivshin, Alyona Lakomkina, Ilya Bezkishko, Aleksandr Kononov, Oleg Sinyashin, Yulia Budnikova, Olga Kataeva, Vasily Miluykov

**Affiliations:** 1 Arbuzov Institute of Organic and Physical Chemistry, FRC Kazan Scientific Center, Russian Academy of Sciences, 8 Arbuzov St., 420088 Kazan, Russia; shekurovruslan@gmail.com (R.S.); tatyanagr@gmail.com (T.G.); zika0527@mail.ru (Z.Y.); alena.lakomkina.00@mail.ru (A.L.); bezkishko@gmail.com (I.B.); kononovsnz97@gmail.com (A.K.); oleg@iopc.ru (O.S.); yulia@iopc.ru (Y.B.); olga-kataeva@yandex.ru (O.K.); miluykov@iopc.ru (V.M.); 2 A.M. Butlerov Chemistry Institute of the Kazan Federal University, Kremlevskaya str. 18, 420008 Kazan, Russia; kamil.ivshin@yandex.ru

**Keywords:** multi-ferrocenyl compounds, phosphorus thioesters, DFT calculations, cyclic voltammetry, tetrathiophosphate

## Abstract

The reaction of triferrocenylthiophosphite with elemental sulfur leads to triferrocenyltetrathiophosphate. The molecule of tetrathiophosphate adopts propeller-like all synclinal-conformation of the ferrocenyl fragments respective to the P=S bond. All ferrocenyl groups have nearly ideal eclipsed conformation of the cyclopentadienyl fragments. The Fc_3_S_3_P (**1**)**,** Fc_3_S_3_P=O, (**2**) and Fc_3_S_3_P=S (**3**) demonstrate three reversible and well-separated ferrocenyl-based redox events. The electronic structures of **1**–**3** have been studied quantum-chemically; the energies and composition of frontier orbitals have been calculated.

## 1. Introduction

Currently, a number of new practical applications of multi-ferrocenyl compounds are being actively developed. [1,2,3,4,5,6,7,8,9,10,11] The multi-ferrocenes are widely used as model systems for understanding intramolecular charge transfer processes. Consequently, more and more multi-ferrocenes are synthesized to study their charge transfer processes and to match intramolecular charge transfer interactions numbers. [3,4,5,6,7,8,9,10,11,12,13] Typically, the multi-ferrocenyl compounds contain the π-conjugated spacers binding two or more ferrocene moieties (see for example [12,13]). The approach based on the construction of multi-ferrocenyl compounds using spacers capable of transmitting exchange interactions was successfully implemented to obtain new practically significant materials. At the same time, the disadvantage of this approach is the complexity of creating multi-ferrocene molecules, since the syntheses proceeds in several stages and, as a rule, with low yields.

An alternative way to multi-ferrocenyl compound is using phosphorus acids derivatives, containing ferrocene fragments. These ferrocene derivatives can be easily accessed via low cost syntheses with good yields [14,15]. It was demonstrated that ferrocene containing phosphate molecules can be successfully used for the construction of multi-step multi-electron redox systems [16,17,18,19]. For example, M. Korb [18,19] and co-workers showed that triferrocenyl phosphate and other P, O-substituted multi-ferrocenyl compounds exhibit one reversible one-electron redox peak for each ferrocene fragment.

However, the electrochemical properties of sulfur-containing analogues have not yet been investigated, albeit such studies are of special importance in relevance to electronic structure and dual reactivity of phosphorus-sulfur ambident system. The reason is that the incomplete thioesters of acids of trivalent phosphorus, in this case, have an additional binding center with a metal ion, which provides a rigid, unstable fixation.

In this respect, cyclic voltammetry (CV) is a very useful technique to evaluate the multi-step multi-electron transfer processes of molecules with multiple redox-active sites. Moreover, the CV measurements of multi-ferrocene derivatives provide important information on the degree of electronic communication between the ferrocene and ferrocenium cation.

Herewith, we report the synthesis and structural properties of new triferrocenyltetratiophosphate in comparison with trivalent phosphorus derivative and triferrocenyltrithiophosphate: Fc_3_S_3_P (**1**), Fc_3_S_3_P=O (**2**), and Fc_3_S_3_P=S (**3**).

## 2. Results and Discussion

**Scheme 1:**
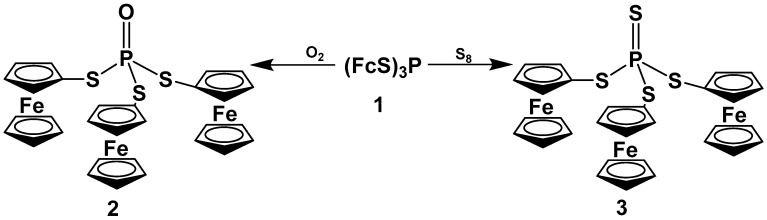
Synthesis of **2** and **3**.

The initial triferrocenyltrithiophosphite **1** and its trithiophosphate derivative **2** were synthesized using the procedures elaborated in our group. [20] Tetrathiophosphate **3** was previously unknown in the literature, as far as we know. In the present study it was obtained via the reaction of triferrocenyltrithiophosphite with elemental sulfur S_8_ in benzene to with good yield. (Scheme 1). The crystallization of the product by slow evaporation from a mixture of a benzene-hexane mixture gave single crystals suitable for X-ray diffraction.

**Figure 1:**
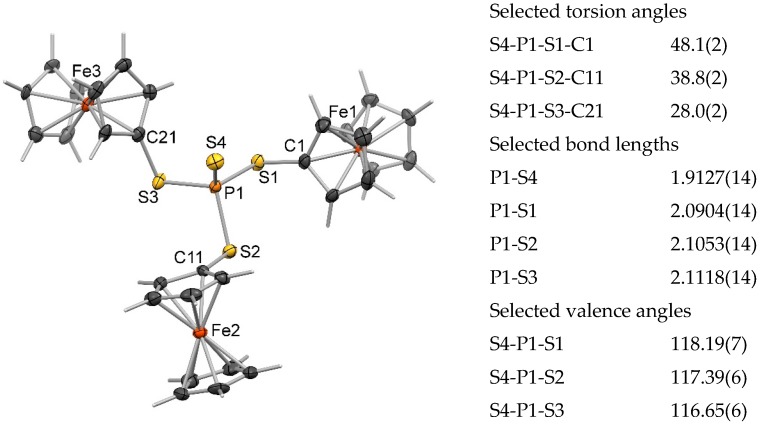
ORTEP drawing of **3** at the 50% probability level and selected geometrical parameters.

The X-ray single crystal diffraction study showed, that the molecule of triferrocenyltetrathiophosphate adopts a propeller-like conformation with synclinal orientation of the ferrocenyl fragments respective to the P=S bond (Figure 1). CCDC 1957135, for compound **3** contain the Supplementary crystallographic data of this paper. All ferrocenyl groups have nearly ideal eclipsed conformation of the cyclopentadienyl fragments, as in triclinic [21] and orthorhombic [22] polymorphic forms of ferrocene itself, while the staggered orientation of the cyclopentadienyl rings being observed in monoclinic [23,24] ferrocene. In a plethora of structures, containing monosubstituted ferrocene fragments, reported in the Crystal Structural Database [25], both eclipsed and staggered orientations are observed with nearly equal occupancy.

Interestingly, one can see that bond lengths and valence angles at phosphorus change in concert with the orientation of ferrocenyl groups, i.e., the smaller is the torsion angle, the larger is the bond length and smaller is the valence angle. As expected, the bond lengths change in shorter limits ranging from 2.0904(14) to 2.1118(14) Å. The valence angles at phosphorus atom range from 116.65(6) to 118.19(7)°, while the valence angles at all sulphur atoms are the same within experimental errors, the average value is equal to 100.4°.

Similar propeller-like structure was observed in crystalline 2 [20] with the corresponding torsion angles ranging from 32° to 44°. One should note that the P-S bonds in 3 (data for 150 K) are longer than in 2 (P-S 2.079–2.090(2) Å at 290K), the difference would be even more significant for the structures collected at the same temperature. The difference between the P-S bonds in the corresponding phenyl containing trithiophosphate (SIXXUV entry in Cambridge Structural Database) [25] and tetrathiophosphate [23], both studied at room temperature, is 0.022 Å. Notably, the P-S bond lengths in triphenyltrithiophosphate (2.075–2.087 Å) are similar as in tripherrocenyltrithiophosphate 2, the same applies to tetraphosphate derivatives: the P-S bonds in triphenyltetrathiophosphate range from 2.099 to 2.103 Å.

This geometry difference is most probably determined by the electronic structure of the P=O and P=S derivatives. The electronic structure, especially the energy levels of frontier molecular orbitals, is essential for the redox properties of the studied molecules. Thus, quantum chemical calculations were performed for a series of multi-ferrocenyl compounds **1**–**3**. The optimized structures show the same trend: P-S is 2.12 Å for **2** compared to 2.14 Å for **3**. It should be mentioned, that for 1 quantum chemical computations predict even longer P-S bonds 2.15 Å.

The formation of P=S or P=O bond leads according to computations to decreasing the energies of both the highest occupied and the lowest unoccupied molecular orbitals (HOMO and LUMO, Table 1). Computations for compound **2** predict slightly lower energies of HOMO and LUMO, with more pronounced effect for the latter, leading to decrease of HOMO-LUMO gap for **3** compared to **2**. HOMOs of compounds **1**–**3** are localized mostly on Fc moieties; however, with participation of phosphorus atomic orbitals for P in **1** and P=S in **3**. LUMOs are mostly contributed by PS_3_ fragments and oxygen, and sulfur atomic orbitals for **2** and **3,** respectively (Table 1).

**Figure 2:**
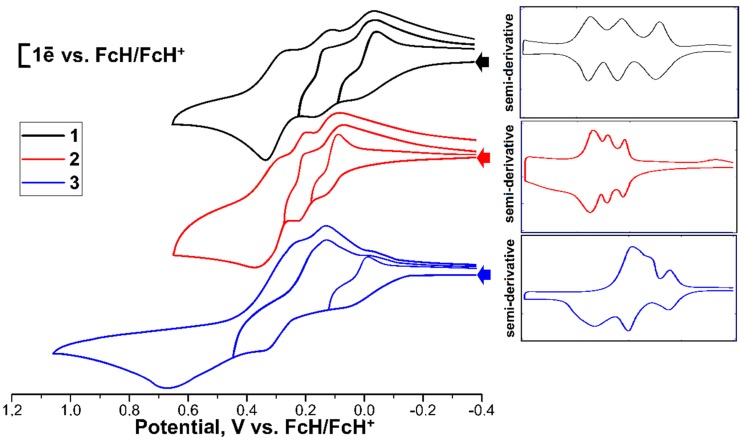
Cyclic voltammograms for oxidation of **1–3**. Conditions: 0.5 mM solutions of **1–3** in CH_3_CN, 0.1 M [NBu_4_][BF_4_]; working electrode: glassy carbon; 10 mVs^−1^, peak potential in V ref. Ag/AgCl recalculated to FcH/FcH^+^.

The cyclic voltammograms of **1**–**3** in a CH_3_CN solution containing 0.1 M [NBu_4_][BF_4_] as the supporting electrolyte showed three well-separated redox events (Figure 2).

It should be noted that **1** and **2** have three classical stepwise reversible transitions of Fe(II) to Fe(III). Cyclic voltammogram of **3** substantially differs in shape from **2** analog. In the case of compound **3**, Ep^2^ and Ep^3^ peak are quasi-reversible. It is interesting to note that the literary compound triferrocenylphosphate (Fc_3_O_3_P=O) is closer in its electrochemical properties to triferrocenyltrithiophosphate (**2**), then **3** differs significantly from both (Table 2).

**Figure 3:**
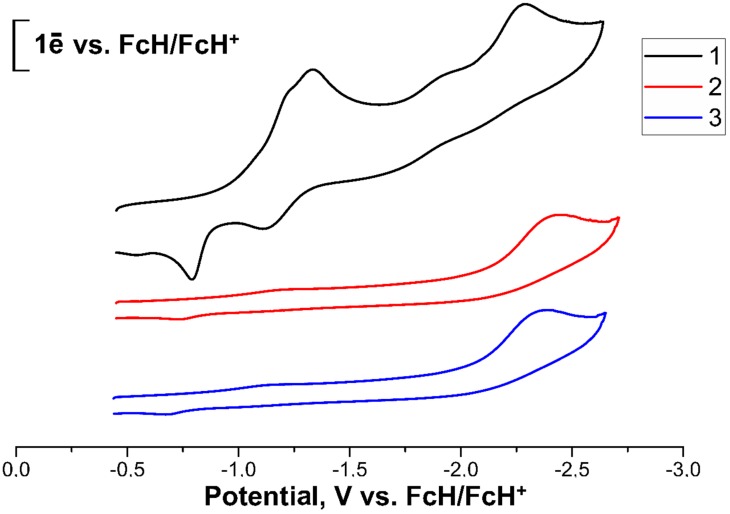
Cyclic voltammograms for reduction of **1–3**. Conditions: 0.5 mM solutions of **1–3** in CH_3_CN, 0.1 M [NBu_4_][BF_4_]; working electrode: glassy carbon, 10 mVs^−1^, peak potential in V ref. Ag/AgCl recalculated to FcH/FcH^+^.

Compounds **1–3** were also studied in the cathodic part. It was found that **1** with a low reduction potential is characterized by an almost synchronous two quasi-reversible one-electron processes. Probably, phosphorus-centered electron transfer occurs with the formation of an anion Fc_3_S_3_P^−^ that is unstable during reverse scanning (Figure 3). The electron transfer between the ferrocenyl substituents in mixed valent oxidation states does not occur, as shown recently [19].

**Figure 4:**
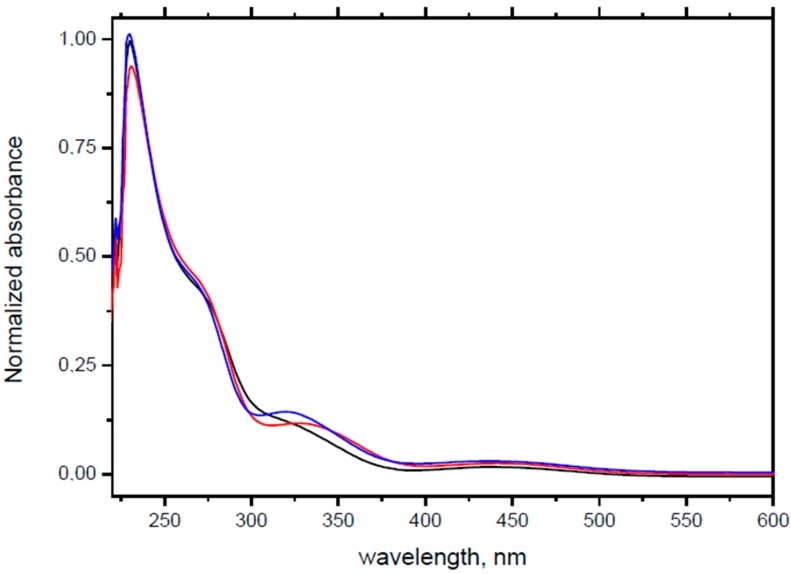
UV/Vis spectra of **1** (black), **2** (blue) and **3** (red) in dichloromethane, c = 10^−5^ mol/L.

The differences in the electronic structures of compounds **1–3** may cause the changes in their spectral properties. UV spectra of the compounds **1–3**, registered in dichloromethane, demonstrate bands at 436–442 nm, 320–327 nm, shoulders at 270 nm and strong bands below 200 nm (Figure 4). The low energy band is most probably related with ferrocene [26]. Interestingly that formation of P=S bond leads to slight bathochromic shift of this band (442 nm) whereas in P=O compound this band is hypsochromically shifted to 436 nm, compared to 438 nm in **1**.

## 3. Materials and Methods

### 3.1. Reagents and Research Subjects

All the work related to the preparation of the initial reagents, the synthesis, and the release of products was carried out in an inert atmosphere using standard Schlenk apparatus. All solvents and purchased reagents Alfa Aesar (Heysham, Lancashire, UK), Merck (Darmstadt, Germany), Sigma-Aldrich (St. Louis, MO, USA) were absolute by the appropriate methods, mainly by distillation in an inert atmosphere. The triferrocenyltrithiophosphite (**1**) and triferrocenyltrithiophosphate (**2**) were obtained according [20].

### 3.2. Synthesis of Triferrocenyltetrathiophosphate (**3**)

A solution of triferrocenyltrithiophosphite (**1**) (2.05 g, 3 mmol) in benzene (20 mL) was boiled with elemental sulfur (0.2 g, excess) during three hours. The solvent was evaporated and the residue was recrystallized from a mixture of benzene/hexane (2:1) to give dark-orange crystals of **3** (1.94 g, 2.7 mmol, 90%), m.p. 237–239 °C. ^1^H NMR (CDCl_3_): δ = 4.39 (m, 2H_α_), 4.50 (m, 2 H_β_), 4.25 (s, 5H); ^31^P{^1^H} (CDCl_3_): δ = 89.7—C_30_H_27_Fe_3_PS_4_ (714.28): C 50.44, H 3.81, P 4.34, S 17.96; found C 50.65, H 3.83, P 4.30, S 17.85

### 3.3. X-ray Crystallography

Data set for single crystal **3** (CCDC 1957135) was collected on a Bruker AXS Kappa Apex II diffractometer (Bruker AXS GmbH, Karlsruhe, Germany) with graphite-monochromated Mo K_α_ radiation (λ = 0.71073 Å). The structure of **3** was solved by direct methods using APEX3 [27] for data collection, SAINT [28] for data reduction, SHELXS [29] for structure solution, SHELXL [29] for structure refinement by full-matrix least-squares against F^2^, and SADABS [30] for multi-scan absorption correction. Hydrogen atoms at carbon atoms were placed into calculated positions and refined as riding atoms. The data collection and refinement parameters are given.

*Crystal **3***: empirical formula C_30_H_27_Fe_3_S_4_P, M = 714.28 g/mol, crystal size (mm) 0.207 × 0.126 × 0.027, temperature 150(2) K, monoclinic, space group *P*2_1_/*c* (No. 14), a = 19.8510(10) Å, b = 11.8071(7) Å, c = 12.8277(7) Å, β = 100.699(4)°, V = 2954.3(3) Å^3^, Z = 4, ρ_calc_ = 1.606 g·cm^−3^, μ = 1.810 mm^−1^, θ range: from 1.04° to 26.00°, 63501 reflection collected (−*22 ≤ h ≤ 23*, −*14 ≤ k ≤ 14*, −*15 ≤ l ≤ 15*), 5721 independent reflections (*R_int_* = 0.1322), 3172 observed reflections with *I ≥ 2σ(I)*, 334 refined parameters, *R* = 0.0384, *wR^2^* = 0.0539, max. Residual electron density 0.406 (−0.401) eÅ^−3^.

### 3.4. NMR Experiments

The NMR spectra were registered on the equipment of Assigned Spectral-Analytical Center of FRC Kazan Scientific Center of RAS, namely on multi-nuclear spectrometer Bruker AVANCE-400 (BRUKER BioSpin GMBH am Silberstreifen, D-76287, Rheinstetten, Germany) (400.1 MHz {^1^H} and 162.0 MHz {^31^P}).

### 3.5. UV/Vis Experiments

Electronic absorption (UV-Vis) spectra were recorded at room temperature on a Perkin-Elmer Lambda 35 spectrometer (PerkinElmer, Inc, Waltham, MA (Massachusetts), USA) using 10 mm quartz cells. Absorption spectra were registered with a scan speed of 480 nm/min, using a spectral width of 1 nm. All samples were prepared as solutions in dichloromethane with the concentrations ~10^−5^ mol L^−1^.

### 3.6. Calculations

All calculations were performed with the Gaussian 16 suite of programs [31]. The hybrid PBE0 functional [32] and the Ahlrichs’ triple-ζ def-TZVP AO basis set [32] were used for optimization of all structures. In all geometry optimizations, the D3 approach [33] to describe the London dispersion interactions together with the Becke–Johnson (BJ) damping function [34,35,36,37] were employed as implemented in the Gaussian 16 program.

### 3.7. Electrochemistry

Cyclic voltammograms were recorded with a BASi Epsilon E2P (USA) potentiostat. The device comprises a measuring unit, PC DellOptiplex 320 (Bioanalytical Systems, Inc., West Lafayette, IN, USA) with the Epsilon-EC-USB-V200 software. Tetrabutylammonium tetrafluoroborate [NBu_4_][BF_4_] was used as background electrolyte. The working electrode was a stationary disc glassy-carbon electrode (the surface area of 6 mm^2^). Ag/AgCl (0.01 M KCl) was used as a reference electrode. Ferrocene was added as an internal standard for Ag/AgCl conversion (E_1/2_ = 0.45V). The reference electrode was connected with the cell solution by a modified Luggin capillary filled with the supporting electrolyte solution (0.1 M Bu_4_NBF_4_ in CH_3_CN). Thus, the reference electrode assembly had two compartments, each terminated with an ultra-fine glass frit to separate the AgCl from the analyte. A platinum wire was used as an auxiliary electrode. The scan rate was 100 mV∙s^−1^. The measurements were performed in a temperature-controlled electrochemical cell (volume from 1 mL to 5 mL) in an inert gas atmosphere (N_2_). Between measurements or prior to a registration of a voltammetry wave, the solution was actively stirred with a magnetic stirrer in the atmosphere of constant inflow of an inert gas that was run through a dehydrating system, and then through a nickel-based purification system BI-GAS cleaner (manufactured by OOO Modern Laboratory Equipment, Novosibirsk, Russia) to remove trace quantities of oxygen.

## 4. Conclusions

A new multi ferrocenyl-substituted compound, triferrocenyltetrathiophosphate, was synthesized and was shown to adopt propeller-like conformation in crystalline form. Its electrochemical properties, as well as of the parent compound triferrocenyltrithiophosphite (**1**) and oxygen containing derivative triferrocenyltrithiophosphate (**2**) were studied and exhibit a number of interesting features.

Three reversible one-electron peaks corresponding to the stepwise oxidation of three ferrocene fragments are observed in the cyclic voltammograms of oxidation of **1**, and the first oxidation potential is almost identical to free ferrocene. A similar behavior is also demonstrated by triferrocenyltrithiophosphate (**2**), in which, however, the peaks are slightly shifted by 140 mV in comparison with the initial triferrocenyltrithiophosphite (**1**). At the same time, the oxidation process of triferrocenyltetratiophosphate (**3**) proceeds differently—in the first stage, reversible one-electron oxidation occurs, while the second and third peaks are quasi-reversible. When triferrocenyltrithiophosphite (**1**) is reduced, a quasi-reversible two-electron transfer is observed related to the formation of a phosphorus anion, which is absent in the case of **2** and **3**.

Structures **1**–**3** were optimized quantum-chemically and the composition and energies of the frontier orbitals were calculated. It was shown that for the studied trithiophosphites, the three upper occupied orbitals have close energies. The HOMOs are localized mainly on ferrocene fragments, while the PS_3_ fragment makes the main contribution to the lower free orbitals. The results of the present work can be used for design of electrooxidized forms of a mixed type of ligands and their complexes with transition metals, possessing interesting magnetic properties.

## Figures and Tables

**Table 1:** Predicted energies of frontier orbitals of optimized compounds **1**–**3**.

HOMO-2.	HOMO-1	HOMO	LUMO	LUMO+1	LUMO+2
Compound **1** (ΔE_HOMO − LUMO_ = 5.24 eV)					
−5.92	−5.92	−5.89	−0.54	−0.48	−0.48
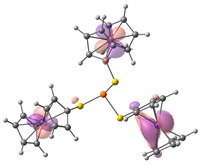	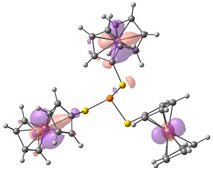	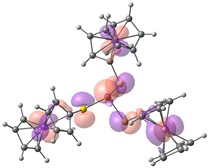	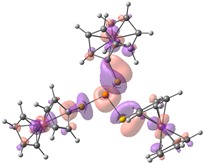	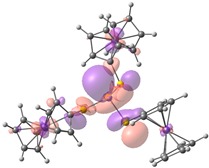	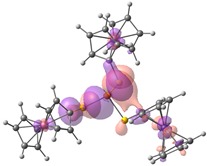
Compound **2** (ΔE_HOMO − LUMO_ = 5.03 eV)					
−5.98	−5.95	−5.93	−0.90	−0.15	−0.14
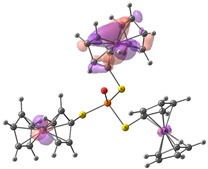	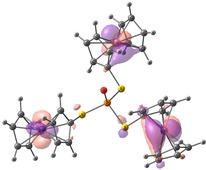	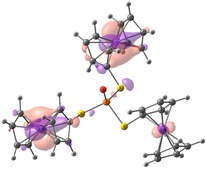	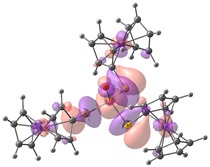	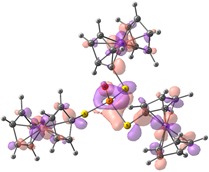	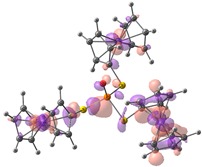
Compound **3** (ΔE_HOMO − LUMO_ = 4.86 eV)					
−5.99	−5.95	−5.95	−1.09	−0.35	−0.34
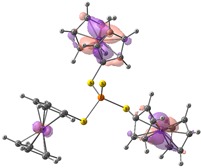	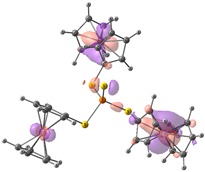	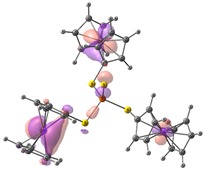	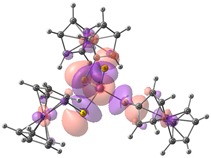	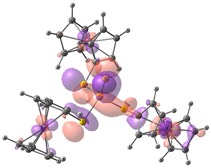	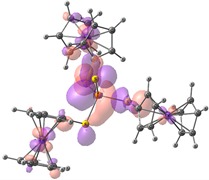

**Table 2:** Electrochemical data for oxidation-reduction of **1–3**. Conditions: 0.5 mM, CH_3_CN, 0.1 M [NBu_4_][BF_4_]; working electrode GC; 10 mVs^−1^, peak potential in V ref. Ag/AgCl recalculated to FcH/FcH^+^; irrev—irreversible, f—forward, b—backscan.

Oxidation	^1^*E*_p_^f^/*E*_p_^b^, V	Δ*E*_p_^f–b^, V	^2^*E*_p_^f^/*E*_p_^b^, V	Δ *E*_p_^f-b^, V	^3^*E*_p_^f^/*E*_p_^b^, V	Δ *E*_p_^f–b^, V
Fc_3_S_3_P (1)	0.00/−0.06	0.06	0.16/0.10	0.06	0.32/0.26	0.06
Fc_3_S_3_P=O (2)	0.14/0.08	0.06	0.23/0.17	0.06	0.35/0.29	0.06
Fc_3_S_3_P=S (3)	0.04/−0.02	0.06	0.33/0.12	0.21	0.64/0.25	0.39
Fc_3_O_3_P=O [19]	0.03/−0.03	0.06	0.24/0.17	0.07	0.44/0.37	0.07
**Reduction**						
Fc_3_S_3_P (1)	−1.23/−0.79	0.44	−1.33/−1.11	0.22	−2.28/irrev	-
Fc_3_S_3_P=O (2)	−2.44/irrev	-				
Fc_3_S_3_P=S (3)	−2.37/irrev	-				

## Supplementary Materials

The following are available online. Supplementary Material 1: checkcif.pdf, Supplementary Material 2: CCDC 1957135.cif.
